# Activation of Piezo1 increases the sensitivity of breast cancer to hyperthermia therapy

**DOI:** 10.1515/med-2024-0898

**Published:** 2024-03-04

**Authors:** Shao-kang Wang, Xiao-ting Zhang, Xuan-yao Jiang, Bi-jiang Geng, Tao-lin Qing, Lei Li, Yun Chen, Jin-feng Li, Xiao-fang Zhang, Shuo-gui Xu, Jiang-bo Zhu, Yu-ping Zhu, Mei-tang Wang, Ji-kuai Chen

**Affiliations:** Department of Emergency, Changhai Hospital, Naval Medical University, Shanghai, 200433, China; Faculty of Anesthesiology, Changhai Hospital, Naval Medical University, Shanghai, China; Department of Health Toxicology, Faculty of Naval Medicine, Naval Medical University, Shanghai, 200433, China; Department of Emergency, The Second Naval Hospital of Southern Theater Command of PLA, Hainan, China; Heatstroke Treatment and Research Center of PLA, Hainan, China; Basic Medical Experimental Teaching Center, Basic Medical College, Naval Medical University, No 800, Xiangyin Road, Shanghai, 200433, China

**Keywords:** photothermal therapy, heat stress, Piezo1, breast cancer

## Abstract

Photothermal therapy (PTT) of nanomaterials is an emerging novel therapeutic strategy for breast cancer. However, there exists an urgent need for appropriate strategies to enhance the antitumor efficacy of PTT and minimize damage to surrounding normal tissues. Piezo1 might be a promising novel photothermal therapeutic target for breast cancer. This study aims to explore the potential role of Piezo1 activation in the hyperthermia therapy of breast cancer cells and investigate the underlying mechanisms. Results showed that the specific agonist of Piezo1 ion channel (Yoda1) aggravated the cell death of breast cancer cells triggered by heat stress *in vitro*. Reactive oxygen species (ROS) production was significantly increased following heat stress, and Yoda1 exacerbated the rise in ROS release. GSK2795039, an inhibitor of NADPH oxidase 2 (NOX2), reversed the Yoda1-mediated aggravation of cellular injury and ROS generation after heat stress. The *in vivo* experiments demonstrate the well photothermal conversion efficiency of TiCN under the 1,064 nm laser irradiation, and Yoda1 increases the sensitivity of breast tumors to PTT in the presence of TiCN. Our study reveals that Piezo1 activation might serve as a photothermal sensitizer for PTT, which may develop as a promising therapeutic strategy for breast cancer.

## 1 Introduction

Breast cancer has become the most threatening cancer. It now ranks first in incidence with an estimated 2.3 million new cases, representing 11.7% of all cancer cases [1]. According to cancer statistics, people who die from breast cancer may mount to 43,780 in the United States in 2022 [2]. Currently, locoregional treatment and systemic therapy remain the mainstay of treatment. However, due to the existence of heterogeneity, many patients are unable to achieve satisfactory outcomes. Current research emphasis is shifting to more biologically directed therapies and reducing the adverse effects of treatment.

Hyperthermia therapy is a new type of adjuvant means of cancer therapy. With the utilization of exogenous heat induction, hyperthermia therapy has considerable potential to augment cancer therapy with minimal toxicity, including inhibiting DNA repair processes, influencing the blood flow of tumors, and causing direct cytotoxicity to cells that are acid and nutrient-deprived [3,4,5]. Photothermal therapy (PTT) is an emerging controllable antitumor technique. It utilizes photothermal convertible agents to generate hyperthermia targeting the site of tumor and ultimately realize the tumor ablation [6,7]. In our previous study, complete breast tumor ablation without recurrence was achieved in mice with the utilization of the photothermal material NIR-II-CDs or their hybrid liposomal formulation [8]. However, due to the limited tissue penetration of light, it may be unrealistic to cure tumors located deeply in the body or tumor with a large volume. Besides, high-temperature induced ablation of tumor under strong laser carries an underlying risk for heating damage of normal cells nearby the tumor. Therefore, PTT is usually the first choice under mild conditions of tumor, in case the possible spread of tumor caused by insufficient heating [9,10], and it is imperative to develop more adjuvant therapies sensitizing PTT [11].

Piezo1, a mechanosensitive ion channel, is widely expressed in various tissues in the human body, especially those exposed to mechanical stimuli, such as the colon, vascular endothelium, lung, and breast [12,13,14]. As a non-selective Ca^2+^-permeable cation channel, it is known to sense various mechanical stresses, including static pressure, shear stress, and membrane stretch [15,16]. Furthermore, increasing evidence revealed that Piezo1 also has important roles in inflammatory and immune regulations [17,18]. Many studies have reported that Piezo1 is associated with many signaling pathways involved in cancer metastasis, such as angiogenesis, cell migration, and proliferation [19,20,21,22]. In breast cancer, high expression of Piezo1 in the primary breast tumor was reported to associate with increased hazard ratio and corresponding shorter overall survival time [23]. Some researchers suggested that Piezo1 is involved in the process of matrix degradation and enhancing the invasive phenotype [24,25,26]. All these have suggested that Piezo1 may be a promising novel therapeutic target for breast cancer. However, no study was conducted to explore the potential role of Piezo1 in heat stress-induced cellular injury. Therefore, further research is necessary to determine whether Piezo1 could be a potential photothermal sensitizer target in hyperthermia therapy.

In this study, we investigated the underlying role of Piezo1 activation in the effects of hyperthermia therapy on breast cancer cells and explored the underlying mechanisms. Our study revealed that Piezo1 activation aggravated the injury of breast cancer cells under the setting of hyperthermia therapy. Mechanistically, the activation of the Piezo1 ion channel significantly increased the expression of NOX2 and reactive oxygen species (ROS), we supposed that Piezo1 is involved in heat stress via the NOX2/ROS signaling pathway. Additionally, the photothermal nanomaterial called TiCN was used as a PTT strategy. The Piezo1-specific agonist Yoda1 enhanced the effect of TiCN on breast tumor ablation under a 1,064 nm laser. Taken together, these results suggest that Piezo1 regionally activation is a potential therapeutic strategy to improve breast cancer.

## 2 Materials and methods

### 2.1 Mice

Female Balb/c nude mice with the age of 3–5 weeks were purchased from Sippr B&K Laboratory Animal Ltd (Shanghai, China). All mice were housed in a room with proper conditions (20–24°C, 40–60% humidity) and a 12 h light/dark cycle. Mice had access to food and water freely. All experimental procedures were approved by the Institutional Animal Ethics Committee of the Navy Medical University according to the Guide for the Care and Use of Laboratory Animals of the National Institutes of Health.

### 2.2 Tumor models

Two million 4T1 cell suspended in PBS (0.1 mL) was subcutaneously injected into the axillary of nude mice to establish a tumor model.

### 2.3 Characterization of TiCN

Transmission electron microscopy (TEM) images of the sample were recorded on a JEM-2100F microscope operated at 200 kV acceleration voltage using super thin carbon films. X-ray diffraction (XRD) patterns were obtained with a Rigaku 18 KW D/max-2550 using Cu Kα radiation. X-ray photoelectron spectroscopy (XPS) measurements were performed using a Kratos Axis Ultra DLD X-ray photoelectron spectrometer. The zeta potential and size of the sample were recorded by a Malvern Zetasizer Nano ZS90 Zeta potential analyzer. UV-vis-NIR absorption spectrum was obtained by Agilent Cary 5000 spectrophotometers. TiCN was purchased by XFNANO Materials Tech Co., Ltd (Jiangsu, China).

### 2.4 Cell culture

The MDA-MB-231 cells, 4T1 cells, and human umbilical vein endothelial cells (HUVECs) were obtained from the Chinese Academy of Sciences (Shanghai, China). The MDA-MB-231 cells and HUVECs were cultured in DMEM high-glucose medium (Gibco, USA) with 10% fetal bovine serum (FBS) (Gibco, USA). 4T1 cells were cultured with RPMI 1640 supplemented with 10% FBS (Gibco, USA). These cells were incubated at 37°C in 5% CO_2_.

### 2.5 Heat treatment

4T1 cells were seeded into a 96-well culture plate (1 × 10^4^/well) or a 6-well culture plate (4 × 10^5^/well) followed by 12 h starvation. Cells were pretreated with Yoda1 (Selleck, China), GSK2795039 (Selleck, China), or catalase (Beyotime, China) for 1 h and then incubated at 37 or 43°C for 5 h. After the heat treatment, the plates were returned to a 37°C incubator for 24 h as recovery. Subsequently, cells were harvested for the following experiments. All incubation temperatures were maintained within ±0.01°C.

As for the experiments of photothermal nanomaterials (TiCN), cells were incubated in a medium with TiCN (100 μg/mL) after 12 h starvation. Then, the cells were given local irradiation with a 1,064 nm laser (0.6 W/cm^2^) for 7 min/well. After the heat treatment, the plates were returned to a 37°C incubator for 24 h and harvested for the following tests.

In the tumor models, 4T1 cells were used to establish the model, 7 days later, 200 µl of photothermal TiCN (500 µg/mL) were intravenously injected into the 4T1 tumor-bearing mice. One day later, the breast tumors of mice were exposed to a 1,064 nm laser (0.6 W/cm^2^) for 10 min, and then, the volumes of the tumors were measured daily. Yoda1 was administrated 1 h before laser exposure (1.5 mg/kg, intraperitoneal injection). Ten days later, the mice were sacrificed and the tumors were harvested for measurement.

### 2.6 Cell viability assays

Cell death and viability were determined by cell counting-8 (CCK-8) assay and live/dead cells assay (Calcein/PI Cell Viability/Cytotoxicity Assay Kit, Beyotime) according to the manufacturer’s instructions. 4T1 cells were seeded into 96-well plates and cultured. After serum starvation for 12 h, the cells were heated at the indicated temperatures and recovered for certain time intervals. After treatment, the medium was removed, 100 μL serum-free medium and 10 μL CCK-8 solution were added to each well, and the cells were incubated for 2 h at 37°C. Optical density at 450 nm was measured with the multifunctional microplate reader (Molecular Devices, USA). The experiments were performed three times. As for the live/dead cells assay, the DMEM containing 5 μL/mL propidium iodide reagent was added into each well, and the medium was incubated for 5 min under dark conditions. The live/dead cell numbers were measured on the machine CYTATION5 (Biotek, USA).

### 2.7 siRNA transfection

After the 4T1 cells had grown to approximately 60% confluence, they were transfected with either the Piezo1 siRNA or scramble siRNA using Lipofectamine 2000 (ThermoFisher Scientific) according to the manufacturer’s guidelines. The sequences of control and Piezo1 siRNAs are as follows: NC: CCTAAGGTTAAGTCGCCCTCG, si-piezo1: GCTATCAGACACCATTTAT. The expression of Piezo1 was confirmed by western blot and quantitative polymerase chain reaction (qPCR) analysis 48 h post-transfection.

### 2.8 Measurement of ROS

4T1 cells were seeded into 96-well plates and treated with Yoda1 or DMSO for 1 h and then subjected to heat treatment under 43°C for 5 h and recovering for 24 h. The culture medium was removed, and then, the cells were washed with PBS. Cells were then incubated for 30 min at 37°C with 10 μM DCFH-DA (Beyotime, China) to detect intracellular ROS levels. The cells were washed three times with serum-free cell culture solution to fully remove DCFH-DA that did not enter the cells. Cells were then put into the spectrophotometer at the excitation wavelength of 488 nm and emission wavelength of 525 nm. In our experiments, cell viability and normalized level of ROS were detected spontaneously. The normalized ROS level was presented with a relative fluorescence intensity (absorbance/number of cells per well).

### 2.9 Probing intracellular calcium

4T1 cells were seeded into a 96-well plate and stained with 2 μM Fura-2 AM and 0.05% Pluronic F127 (Yeasen Biotechnology, China) for 30 min in an incubator according to the manufacturer’s guidelines. Cells were washed two times in HEPES. Cells were then put into the spectrophotometer which has its heating elements. The cells were subjected to heat treatment at 43°C for 1 h and the intracellular calcium flux was detected dynamically at 2 min intervals. Changes in intracellular Ca^2+^ concentrations were expressed as ratios of Fura-2 fluorescence with excitation wavelengths of 340 and 380 nm and an emission wavelength of 510 nm. The fluorescent intensity of Fura‐2 was measured by a dual‐excitation wavelength method (340/380 nm) with the spectrophotometer.

### 2.10 Western blot

Cell proteins were extracted using RIPA lysis reagent containing protease inhibitors and phosphatase inhibitors, and then, protein quantification was performed using the BCA protein quantification method to calculate protein concentration. After 10% sodium dodecylsulfate-polyacrylamide gel electrophoresis, the protein was transferred to PVDF membranes by eBlot L1 membrane transfer machine (Genscript, Shanghai, China). The protein was sealed with blocking solution at room temperature for 1 h and then added the diluted antibody, anti-Piezo1 (1:300; ab129068), anti-NOX2(1:1000; ab129068), and anti-GAPDH (1:1000; Servicebio) were added and incubated overnight at 4°C. The next day, after washing with TBST three times (10 min/time), an HRP-labeled antibody (1:8000; ICLab, Shanghai, China) was added and incubated for 2 h at room temperature and washed with TBST three times (10 min/time). The proteins were visualized by the machine of GE Amersham AI600 (GE Healthcare, USA).

### 2.11 RNA isolation and qPCR

Total cellular RNA was extracted with TRIzol reagent (Vazyme Biotech, China), reverse-transcribed into cDNA by reverse transcriptase and oligo (dT) primers (Vazyme Biotech, China), and then amplified in a Real-time PCR Detection System with SYBR Green master mix and specific primers. Gene expression was normalized to Gapdh using the 2^−ΔΔCT^ method. The results were from three independent experiments performed in triplicate. Primers were purchased from Sangon Biotech, China. The primer sequences used were listed: Piezo1-F: 5′-CCCTGTTACGCTTCAATGCT-3′, Piezo1-R: 5′-GCTACCGTTTTGTCCCAGAA-3′; Gapdh-F:5′-AGGTCGGTGTGAACGGATTTG-3′, Gapdh-R 5′-TGTAGACCATGTAGTTGAGGT-3′.

### 2.12 Statistical analysis

All data are representative of at least three independent experiments. All statistical analyses were performed using the GraphPad Prism 8.3.0 software. The data were presented as the mean ± standard error of the mean (SEM) as indicated in the legends. The one-way analysis of variance was performed for comparing three or more groups. The two-tailed unpaired Student’s *t*-test was used for comparisons between the two groups. A *P* value of <0.05 was considered statistically significant.

## 3 Results

### 3.1 Piezo1 is highly expressed in 4T1 cells and its agonist Yoda1 aggravates heat-induced injury

**Figure 1:**
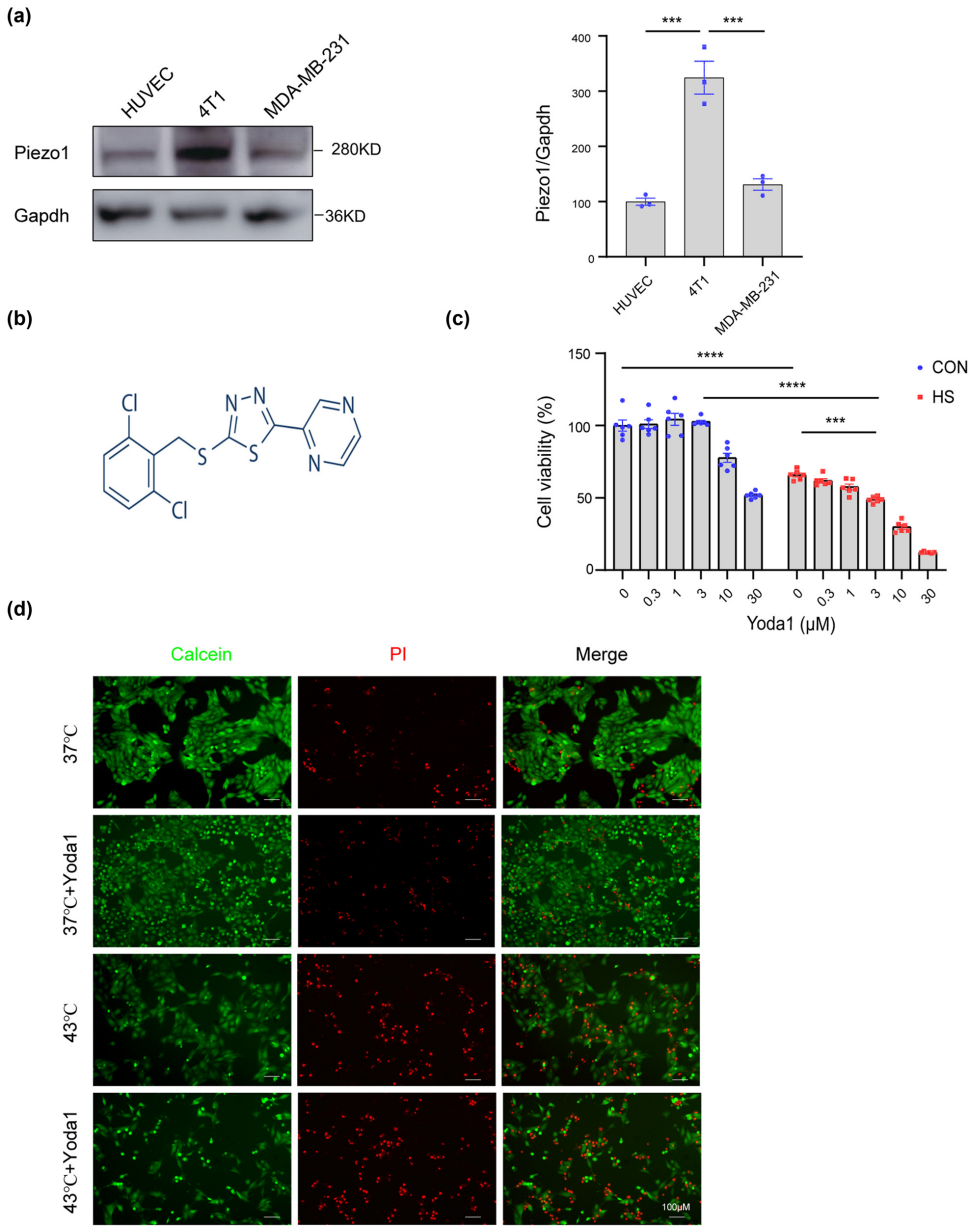
Piezo1 activation aggravates the heat-induced injury. (a) Representative western blotting result of Piezo1 proteins immunoprecipitated from HUVEC, 4T1 cell, and MDA-MD-231 cell using the anti-Piezo1 antibody. The GAPDH level was used for loading control. (b) Structural formula of Yoda1. (c) Cell viability of 4T1 cells pretreated with Yoda1 (0, 0.3, 1, 3, 10, 30 μM) for 1 h and then subjected to heat treatment under 43°C for 5 h and recovering for 24 h. (d) Representative fluorescence images of LIVE/DEAD viability of 4T1 cells pretreated with Yoda1 (3 μM) for 1 h and then subjected to heat treatment under 43°C for 5 h and recovering for 24 h. Alive cells were stained with Calcein (green) and dead cells were stained with PI (red), respectively. The values presented are mean ± SEM (*n* = 6 for each group; ***P < 0.001, ****P < 0.0001, one-way analysis of variance).

Previous studies have reported that Piezo1 is expressed in breast cancer cells. To investigate the potential role of Piezo1 in breast cancer, we first examined the expression of Piezo1 in various breast cancer cells. HUVEC was established as the control. The western blot analysis suggested that Piezo1 had a moderate expression in MDA-MB-231 cells and high expression in the 4T1 breast cancer cells (Figure 1a), indicating the potential role of the Piezo1 ion channel in breast cancer.

To investigate the functional role of the Piezo1 in breast cancer, 4T1 cells were subjected to heat treatment under 43°C for 5 h. It was observed that the cell viability decreased significantly after heat stress. With the pretreatment of Yoda1 (Figure 1b), a specific agonist of Piezo1, the cell death triggered by heat stress was significantly aggravated in a dose-dependent manner (Figure 1c). Given that the cytotoxicity was observed at the high concentration of Yoda1 (10 and 30 μM), 3 μM Yoda1 was finally selected in the subsequent experiments. To visually investigate the survival of breast cancer cells with the treatment of Yoda1, we compared the number of live/dead breast cancer cells with/without 3 μM Yoda1 with a fluorescence microscope. As is shown in Figure 1d, the number of dead 4T1 cells increased significantly under heat stress, and Yoda1 further exacerbated the heat-induced cell death. The result was consistent with that measured by the CCK-8 assay. Altogether, these results indicated the potential role of Piezo1 activation in heat stress-induced breast cancer cell injury.

### 3.2 Yoda1 aggravates heat-induced injury in 4T1 cells through Piezo1 activation

**Figure 2:**
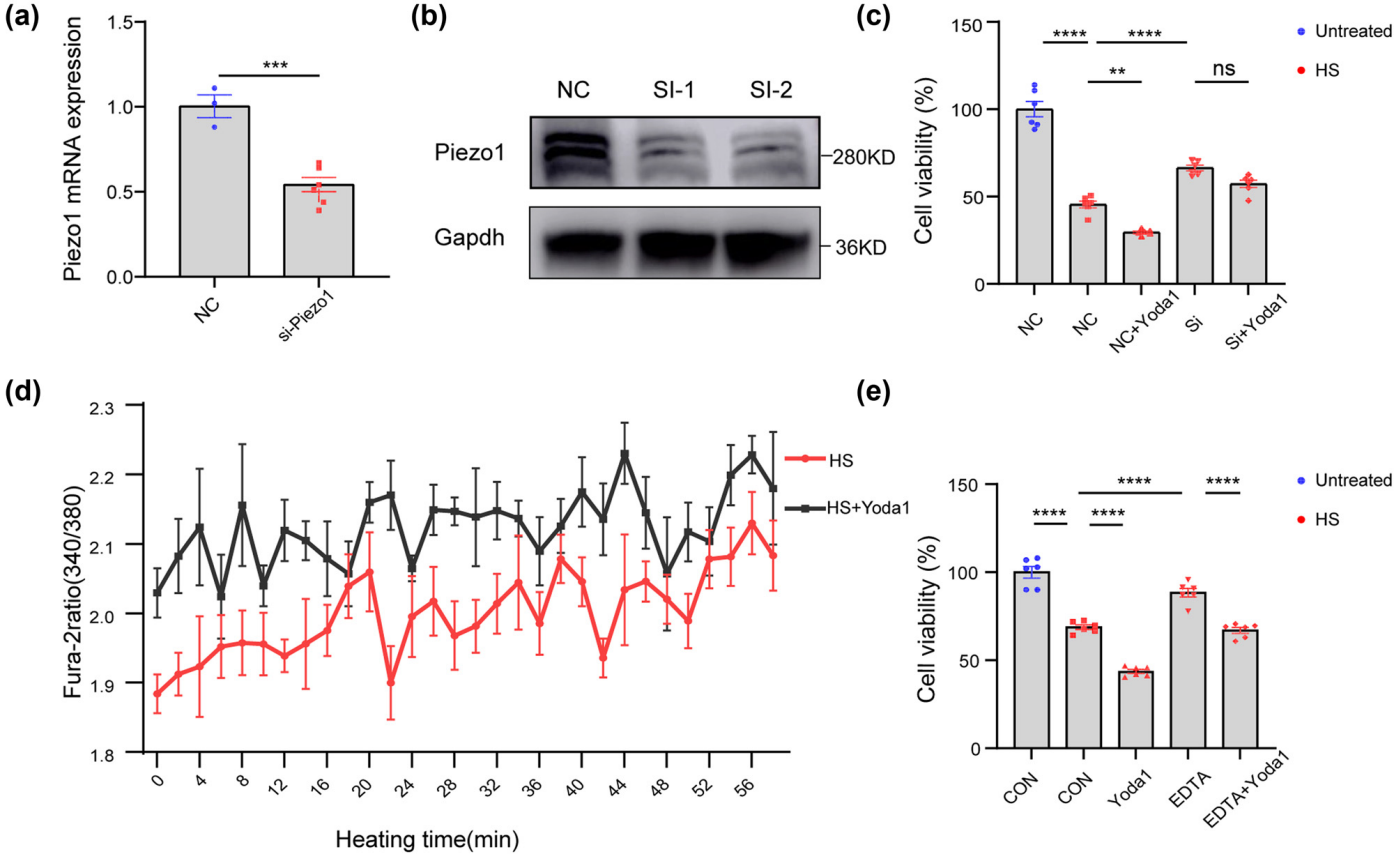
Yoda1 aggravates heat-induced injury in 4T1 cells through Piezo1 activation. (a) The relative mRNA level of Piezo1 in Piezo1 siRNA-transfected 4T1 cells was detected by real-time polymerase chain reaction. (b) Representative western blotting image of Piezo1 proteins in Piezo1 siRNA-transfected 4T1 cells. (c) Cell viability of 4T1 cells pretreated with Yoda1 (3 μM) or transfected with Piezo1 siRNA and then subjected to heat treatment under 43°C for 5 h and recovering for 24 h. (d) Representative average traces of Fura-2 Ca^2+^ imaging of 4T1 cells (6 wells) or Yoda1 pretreated 4T1 cells (6 wells) in response to heat treatment under 43°C for 1 h. (e) Cell viability of 4T1 cells pretreated with Yoda1 (3 μM) or EDTA (1.8 μM) and then subjected to heat treatment under 43°C for 5 h and recovering for 24 h. The values presented are mean ± SEM (*n* = 6 for each group; **P < 0.01, ****P < 0.0001, one-way analysis of variance).

To further investigate the role of Piezo1 in heat stress, 4T1 cells were transfected with siRNA against Piezo1. The expression of Piezo1 was significantly reduced at both mRNA and protein levels (Figure 2a and b), indicating that the level of Piezo1 in 4T1 cells was successfully knocked down. Cells activated with Yoda1 or/and silenced with Piezo1 siRNA were exposed to heat treatment under 43°C for 5 h. It was observed that Piezo1 knockdown alleviates the capacity of Yoda1 to aggravate heat-induced injury. (Figure 2c). These results further demonstrated that Yoda1 aggravates heat-induced breast cancer cell injury through Piezo1 activation.

We next set up to understand how Piezo1 might affect the heat-induced injury in 4T1 cells. Given that Piezo1 is an essential mechanosensitive cation channel that mediates calcium signaling, it prompted us to further assess the role of Piezo1 in regulating Ca^2+^ homeostasis and heat-induced injury. It was reported that intracellular Ca^2+^ increased under heat stress, which may destroy intracellular homeostasis and aggravate cell damage. However, it remains unknown whether Piezo1-induced Ca^2+^ signals are associated with heat-induced injury. To assess the real-time calcium signaling, 4T1 cells were pretreated with Fura-2 AM and directly heated in the spectrophotometer under 43°C for 1 h. Interestingly, the intracellular Ca^2+^ concentration fluctuated with the heating time, presenting an upward trend (Figure 2d). Remarkably, Yoda1 significantly increased the intracellular Ca^2+^ concentration of the heated 4T1 cells, indicating the essential role of Piezo1-mediated calcium signaling in heat-induced injury. To further assess the role of the increased calcium flux, EDTA was used to reduce free Ca^2+^ in the culture media. It was observed that the chelation of extracellular free Ca^2+^ with EDTA (1.8 μM) significantly inhibited heat-induced cellular injury (Figure 2e). However, Yoda1-mediated cellular injury remains with the existence of EDTA, which may indicate that intracellular calcium homeostasis is also involved in the aggravation effect, given that Piezo1 is highly expressed in the endoplasmic reticulum (ER). Altogether, these results indicated that Yoda1 aggravates heat-induced injury through increased calcium flux.

### 3.3 Piezo1 mediates the heat-induced injury in 4T1 cells through NOX2-ROS signaling pathway

**Figure 3:**
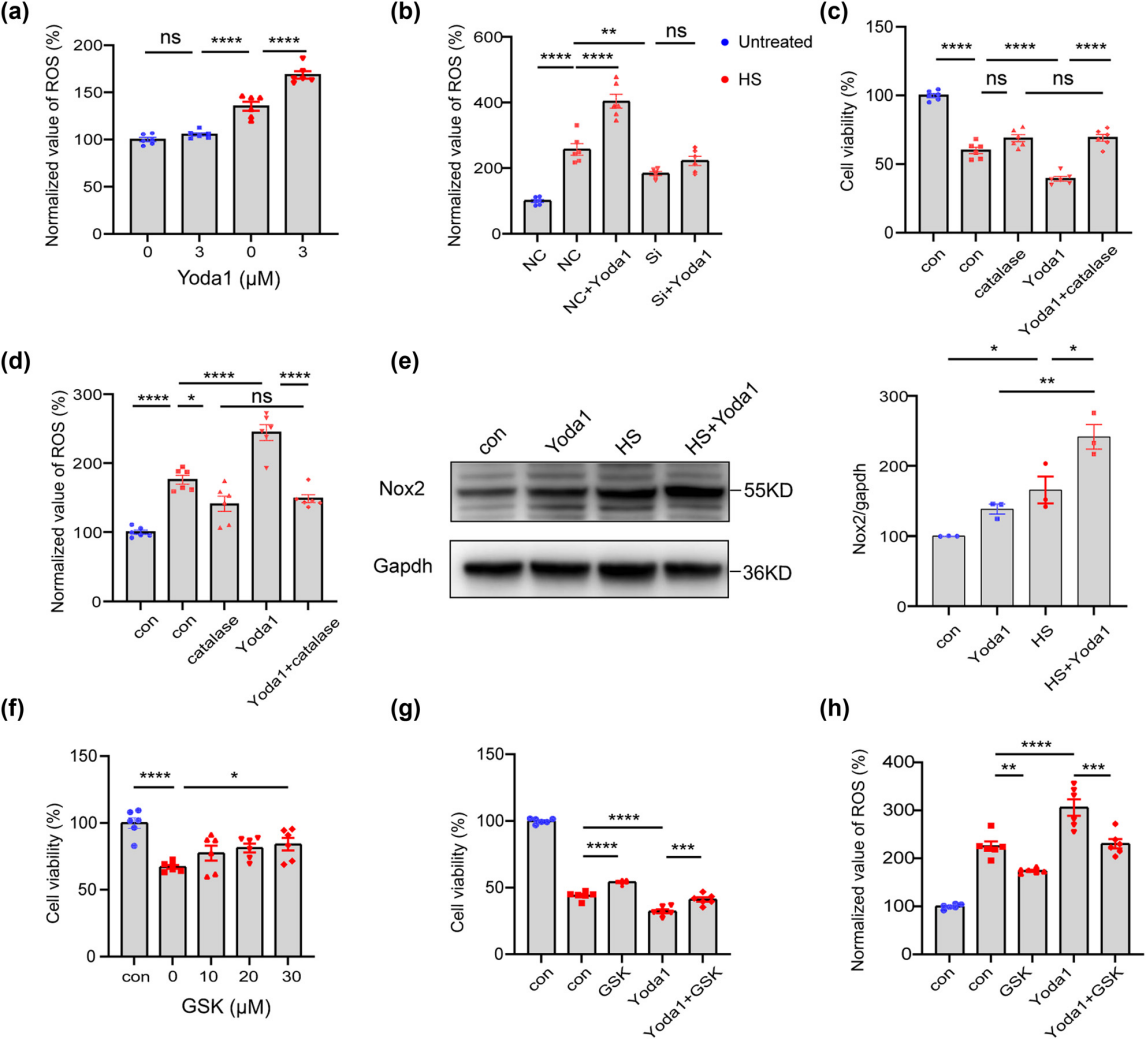
Piezo1 mediates the heat-induced injury in 4T1 cells through the NOX2-ROS signaling pathway. (a) Bar plot of normalized level of ROS in 4T1 cells before and after administration of Yoda1 in the presence or absence of heat treatment under 43°C for 5 h and recovery for 24 h. (b) Bar plot of normalized level of ROS in 4T1 cells pretreated with Yoda1 (3 μM) or transfected with Piezo1 siRNA and then subjected to heat treatment. (c and d) 4T1 cells were pretreated with catalase (300 μg/mL) or Yoda1 (3 μM) and then subjected to heat treatment. Cell viability and normalized level of ROS were detected. (e) Representative western blotting result of NOX2 proteins in 4T1 cells pretreated with Yoda1 (3 μM) and then subjected to heat treatment. (f) Cell viability of 4T1 cells pretreated with GSK2795039 (0, 10, 20, 30 μM) and then subjected to heat treatment under 43°C for 5 h and recovering for 24 h. (g and h) 4T1 cells were pretreated with GSK2795039 (30 μM) or Yoda1 (3 μM) and then subjected to heat treatment. Cell viability and normalized level of ROS were detected. The values presented are mean ± SEM (*n* = 6 for each group; *P < 0.05, **P < 0.01, ***P < 0.001, ****P < 0.0001, one-way analysis of variance).

ROS are byproducts of aerobic metabolism during cellular respiration. An imbalance between ROS generation and the available antioxidant defense against them can lead to oxidative stress. Oxidative stress has been linked to heat stress in animals. However, the potential involvement of Piezo1 in the ROS signaling pathway in the heat-induced damage to 4T1 cells remains unknown. To test this hypothesis, we measured ROS generation in 4T1 cells using the DCFH-DA. Following heat stress, ROS production was observed to increase considerably, while Yoda1 exacerbated the rise in ROS after heat stress (Figure 3a). Furthermore, Piezo1 knockdown significantly alleviated the heat-induced ROS elevation (Figure 3b). These data provide compelling genetic evidence that Piezo1 is involved in inducing ROS release in the setting of heat stress. To further assess the role of ROS signaling in heat stress, the ROS scavenger catalase was administrated. Remarkably, the Yoda1-mediated aggravation of heat stress was significantly reversed with the use of catalase, and both the impaired cell activity and the rise of ROS production were reversed (Figure 3c and d). These results suggested that ROS signaling is essential for heat-induced injury in 4T1 cells, and the Yoda1-induced aggravation can be partly attributed to promoting ROS release.

NADPH oxidase 2 (NOX2), a superoxide-generating enzyme, was identified as one of the key sources of ROS generation. For testing the role of NOX2, the western blot of the 4T1 cells was performed. It was observed that the protein level of NOX2 increased with exposure to heat stress, and it was further enhanced with the treatment of Yoda1 (Figure 3e). GSK2795039, an inhibitor of NOX2, which can competitively inhibit the substrate of NOX2 and the production of ROS. The cell vitality test revealed that GSK2795039 alleviated the heat-induced damage to 4T1 cells in a dose-dependent manner (Figure 3f). GSK2795039 also reversed the Yoda1-mediated aggravation of cellular injury and ROS generation after heat stress (Figure 3g and h). Altogether, these data collectively demonstrate that Piezo1 activation might mediate the heat-induced injury in 4T1 cells through the NOX2-ROS signaling pathway.

### 3.4 Structural characterization of TiCN

**Figure 4:**
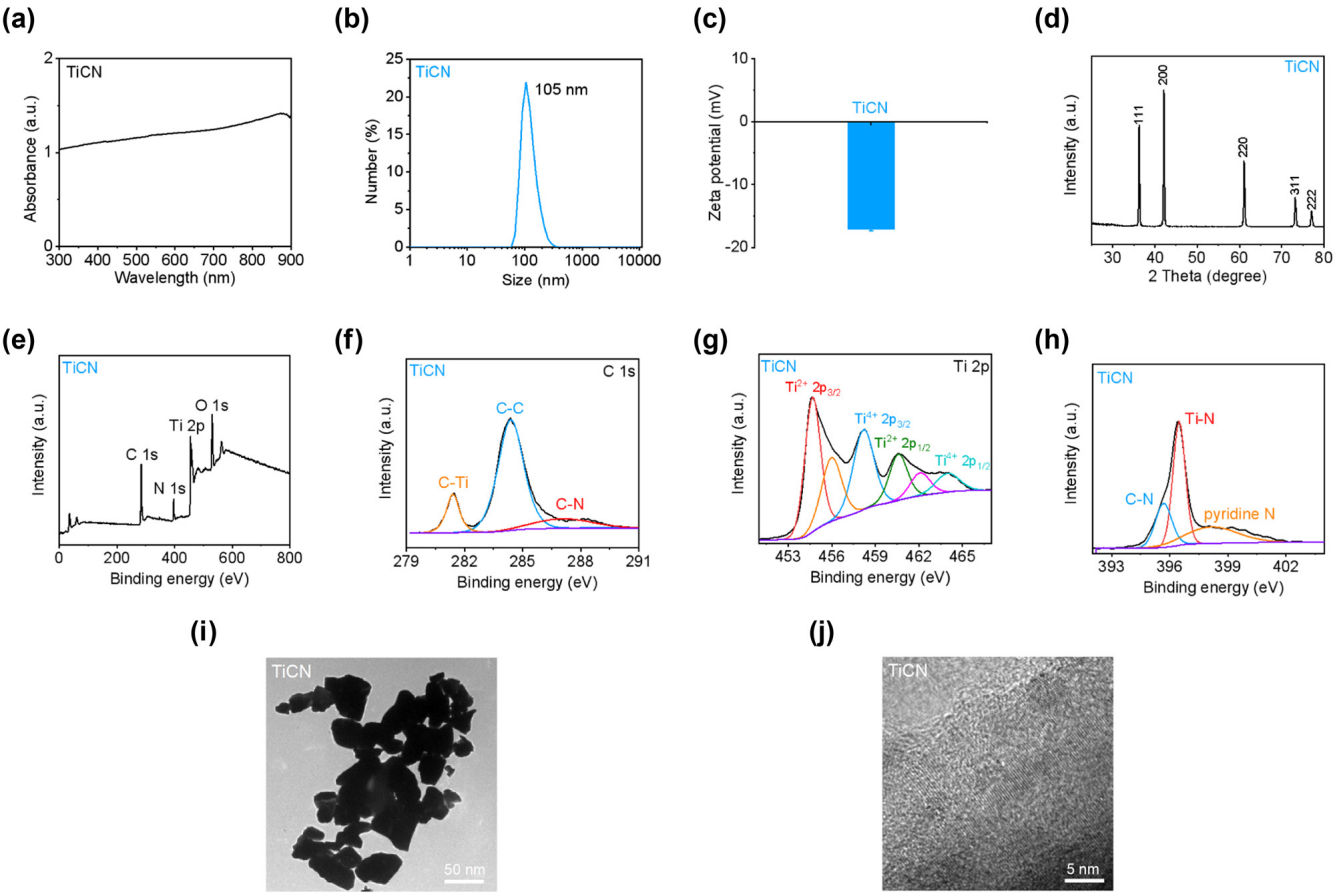
Structural characterization of TiCN. (a) Absorption spectrum of TiCN. (b) Hydrodynamic diameter of TiCN. (c) Zeta potential of TiCN. (d) XRD pattern of TiCN. (e–h) Survey XPS spectrum. High-resolution C 1s, Ti 2p, and N 1s spectra of TiCN. (i and j) TEM and HRTEM images of TiCN.

TiCN is a novel and promising nanomaterial for the application of hyperthermia therapy. The microstructure of TiCN nanosheets was investigated by TEM, XRD, and XPS. After HF etching from bulk TiCN ceramics, the as-synthesized TiCN was in the form of nanosheets. TiCN nanosheets showed a broad absorbance in wavelength of 300–900 nm (Figure 4a). The hydrodynamic diameter of TiCN nanosheets determined by DLS was 105 ± 2.2 nm, which was similar to the TEM results (Figure 4i). Furthermore, the zeta potential of TiCN nanosheets was determined to be −17 ± 0.5 mV (Figure 4c), which could be ascribed to the presence of hydroxyl on the surface of TiCN nanosheets. The XRD pattern of TiCN nanosheets exhibited five dominant peaks at approximately 36, 42, 61, 73, and 77°, corresponding to the (111), (200), (220), (311), and (222) planes of TiCN, respectively (Figure 4d). XPS analysis revealed that TiCN nanosheets contained C, N, Ti, and O elements (Figure 4e), indicating the successful preparation of TiCN nanosheets. C–Ti, C–C, and C–N were presented in the high-resolution C 1s spectrum (Figure 4f). The Ti-containing species of Ti^2+^ 2p_3/2_, Ti^4+^ 2p_3/2_, Ti^2+^ 2p_1/2_, and Ti^4+^ 2p_3/2_ were observed in the high-resolution Ti 2p spectrum (Figure 4g), suggesting the presence of Ti^2+^ and Ti^4+^ in TiCN nanosheets. Moreover, C–N, Ti–N, and pyridine N can be detected in the high-resolution N 1s spectrum (Figure 4h). The HRTEM image presented in Figure 4j demonstrated the high crystallinity of well-dispersed TiCN nanosheets with distinct lattice fringe.

### 3.5 Piezo1 activation has the potential to increase the sensitivity of breast cancer cells to hyperthermia therapy mediated by a novel photothermal nanomaterial

**Figure 5:**
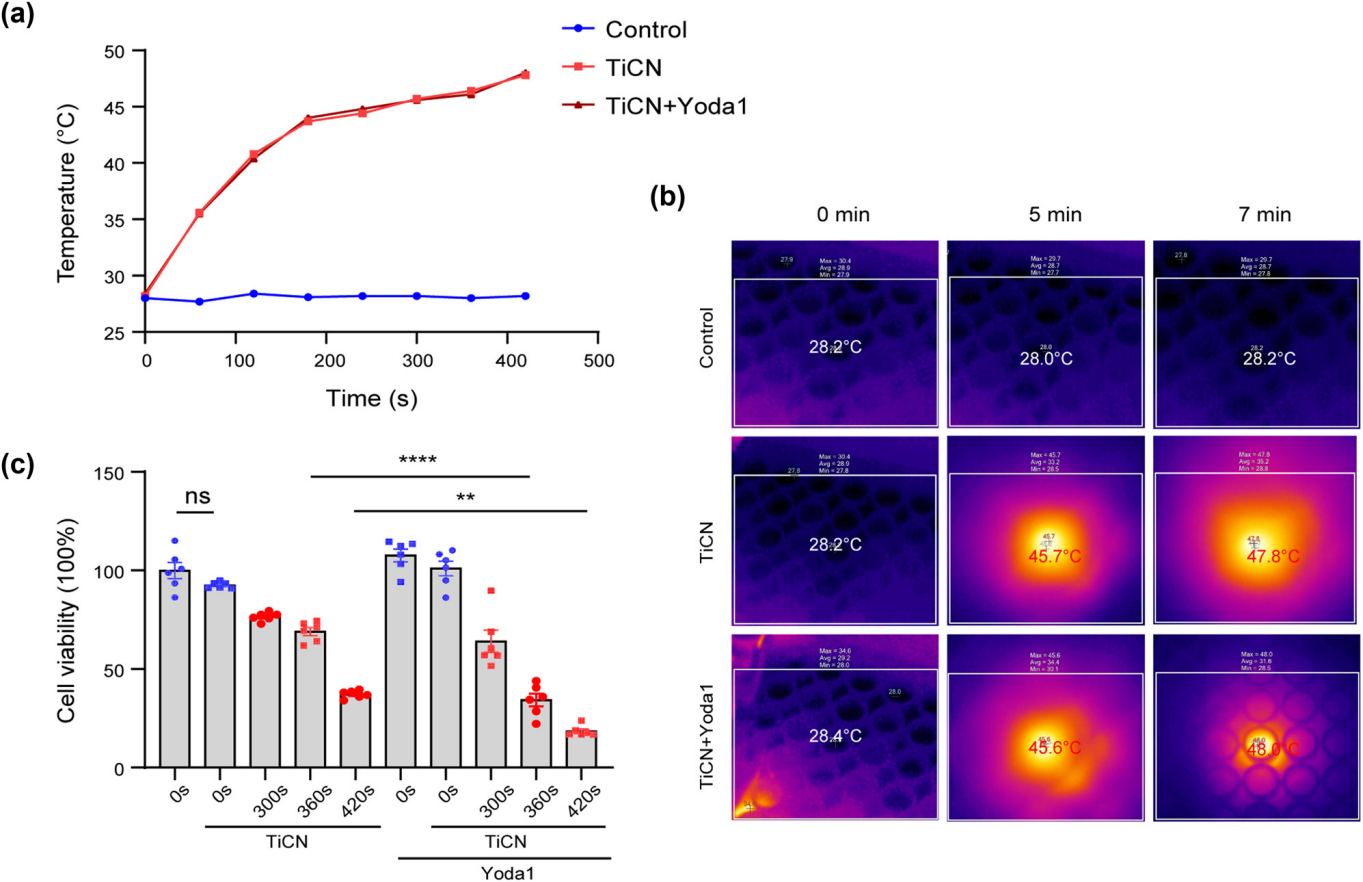
Piezo1 activation has potential in increasing the sensitivity of breast cancer cells to photothermal therapy *in vitro*. (a) Photothermal effect of the TiCN under the 1,064 nm laser at five-ampere. The lasers were shut off after 420 s irradiation. (b) Maximum temperature within the 96-well plates recorded by a thermal imager after a certain time of exposure to a 1,064 nm laser. (c) 4T1 cells were pretreated with TiCN in the presence or absence of Yoda1 (3 μM) and then subjected to heat treatment of 1,064 nm laser for certain durations. 4T1 cells were sent back to a 37°C incubator and then cell viability was detected. The values presented are mean ± SEM (*n* = 6 for each group; ^ns^ *P*＞0.05, **P < 0.01, ****P < 0.0001, one-way analysis of variance).

4T1 cells were seeded into 96-well plates and incubated in a medium with TiCN (100 μg/mL) for 1 h. The cells were then treated with a 1,064 nm laser (0.6 W/cm^2^) for 7 min, and the effects of the photothermal conversion of the nanomaterials were detected with a thermal imager. The ambient temperature of cell culture rapidly ascends within the first 3 min and eventually reaches 48°C at 7 min after a plateau period (Figure 5a and b). After the heat treatment of 7 min, the plates were returned to a 37°C incubator for 24 h and the cell viability was measured. As is shown in Figure 5c, the application of nanomaterials had no toxic damage to cells. The cell viability gradually decreased with the prolongation of the laser irradiation and reduced to 37.18% at 7 min. Furthermore, cell viability decreased to 18.47% in the group of Yoda1 administration. These results suggested the successful establishment of TiCN and the potential sensitizing effects of Yoda1.

### 3.6 Piezo1 activation increases the sensitivity of breast cancer cells to hyperthermia therapy in mice

**Figure 6:**
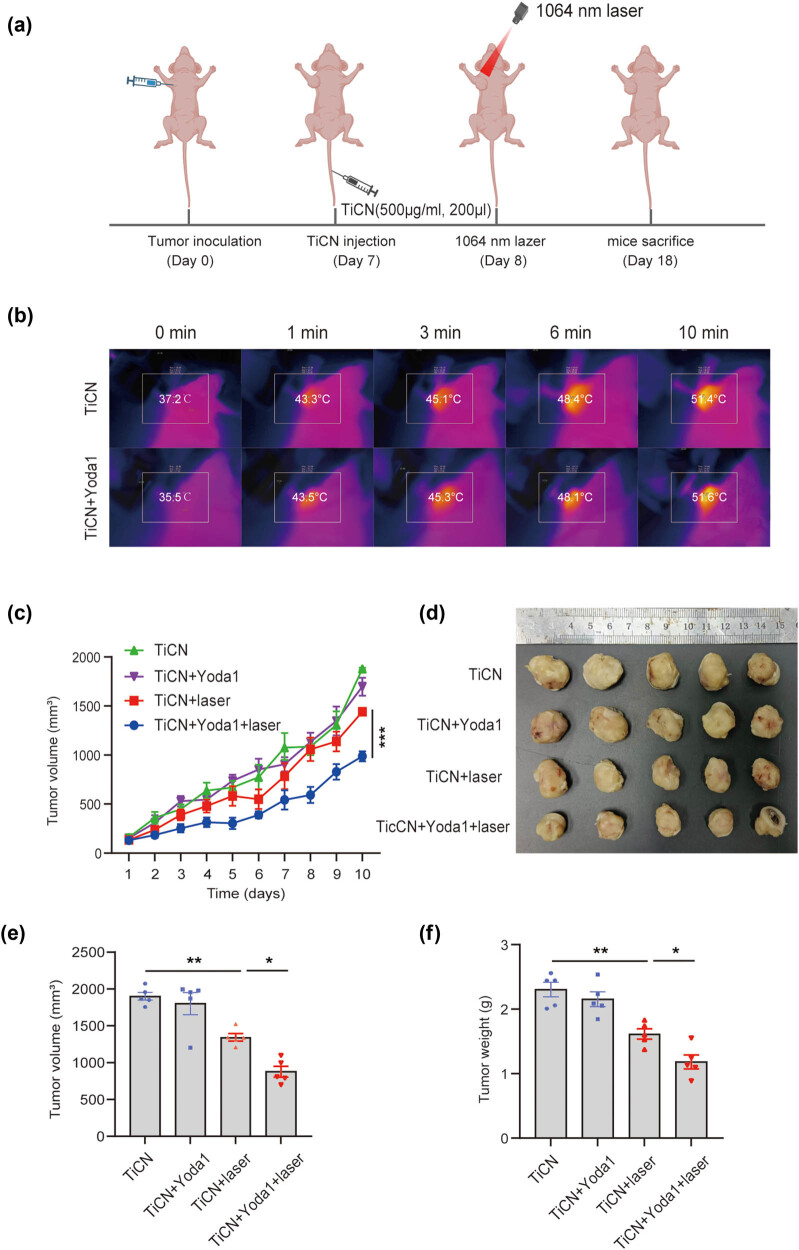
Piezo1 activation increases the sensitivity of breast cancer cells to hyperthermia therapy in mice. (a) Schematic illustration of TiCN-mediated photothermal therapy and administration of Yoda1 for 4T1 tumor-bearing mice. (b) Maximum temperature within the tumor region was recorded by a thermal imager after local administration of 1,064 nm laser irradiation at the power density of 0.6 W/cm^2^. (c) Tumor growth curves of mice after receiving different treatments, including TiCN, TiCN + Yoda1, TiCN + laser, and TiCN + Yoda1 + laser. (d) Pictures of tumors obtained from mice on day 10 after receiving different treatments. (e) Tumor volume and (f) tumor weight of mice were recorded on day 10 after receiving different treatments. The values presented are mean ± SEM (*n* = 5 for each group; *P < 0.05, **P < 0.01, one-way analysis of variance).

In the tumor models, TiCN solutions were intravenously injected into the 4T1 tumor-bearing mice via the tail vein, and the breast tumors of mice were exposed to a 1,064 nm laser (0.6 W/cm^2^) 1 day later. The effects of the photothermal conversion of the nanomaterials were detected with a thermal imager, and the representative images of the local temperature of tumors are shown in Figure 6b. After 10 min hyperthermia therapy, the mice were returned to cages, and the tumor growth curves were recorded for 10 days (Figure 6c). The complete tumor ablation was determined eventually (Figure 6d–f). In mice administrated with nanomaterials, the growth of tumors was significantly reduced after laser irradiation. Although Yoda1 alone did not change the tumor growth, it further enhanced the slowdown of tumor growth in the presence of TiCN.

## 4 Discussion

Piezo1 is an essential mechanical-sensitive ion channel. Through its unique three-blade propeller-shape architecture, Piezo1 transduces various mechanical stimuli into cellular signals via cation influx, mainly Ca^2+^ [12,27]. Yoda1 is the specific agonist of Piezo1, which can effectively lower the channel’s mechanical threshold for activation and trigger Ca^2+^ influx. Transient receptor potential vanilloid 1 (TRPV1), a capsaicin receptor, was previously reported to be activated and open the channel under heat stress. The entry of substantial extracellular Ca^2+^ into the cell consequently caused cell damage [28,29]. However, as a Ca^2+^-permeable channel, Piezo1 has not been reported for its potential role in heat stress. In the current study, the breast cancer cells, 4T1 cells, were transfected by Piezo1 siRNA or administrated with the Piezo1 agonist Yoda1, followed by exposure to heat stress. The results showed that Piezo1 could aggregate the heat-induced injury of 4T1 cells, indicating that Piezo1 can be a promising target for the treatment of breast cancer with heat therapy. This provoked us to think about the role of Piezo1 in the perception of cells to heat stress. We observed that the morphology of some 4T1 cells changed from fibrous to round after the hit of heat stress. We supposed that heat changed the shape of cells and thus brought changes in mechanical forces of cells. Just as the principle of thermal expansion and cold contraction, the deformation of cell membranes was induced by heat and led to the activation of Piezo1.

Ca^2+^ homeostasis plays a critical role in the normal function of cells and tissues. Once an imbalanced homeostasis occurs due to excessive Ca^2+^ influx, cells can come to death. For instance, under the stimulation of noxious heat, the TRPV1 channel is activated by the increases in temperature, and the entry of extracellular Ca^2+^ ensues, which leads to the imbalance of Ca^2+^ homeostasis and cell damage [30,31]. Ryanodine receptor 1 (RyR1), the skeletal muscle ryanodine receptor, is a Ca^2+^-release channel located in the sarcoplasmic reticulum. Its mutation is known to be responsible for the pathogenesis of malignant hyperthermia (MH) [32]. The RYR1-selective inhibitor, Compound 1(Cpd1), effectively prevented and treated the MH and heat stress-induced cellular damage [33]. In addition, the susceptibility to exertional heat stroke is related to the mutation of the *RYR1* gene [33,34,35,36]. Mechanically, heat stress can cause the leakage of Ca^2+^ in the sarcoplasmic reticulum and decrease the ability of the sarcoplasmic reticulum to recover Ca^2+^ [37,38]. Our current experimental results showed that heat stress indeed induced elevated intracellular Ca^2+^ concentration. Strikingly, the Piezo1 agonist Yoda1 further increased the concentration of intracellular Ca^2+^. So, we hypothesized that Yoda1 may aggravate the heat-induced injury by increasing the Ca^2+^ influx. Ca^2+^ -free medium was then used for further experiments. Interestingly, the group of Yoda1 still showed an exacerbated injury, which was though partly alleviated. The results suggested that, apart from the Ca^2+^ homeostasis sustained by the internal and external balance, self-regulation of cells is also of great importance, especially the Ca^2+^ homeostasis in the ER. ER is known as an important Ca^2+^ pool. Under the stimulation of heat stress, ER stress can be triggered due to the imbalanced Ca^2+^ homeostasis and substantial misfolded proteins in the ER, which can eventually cause cell apoptosis [39]. Notably, Piezo1 is also highly expressed in the ER. The exact effect of Yoda1 on Ca^2+^ deserves further investigation. In general, Piezo1 is pivotal in the stability of intracellular Ca^2+^ under heat stress. Yoda1 disrupts the balance and aggravates cell death.

ROS is known to play essential roles in cell physiology and participate in many pathological processes. In the malignant microenvironment, ROS exerts multiple actions in the growth and death during different periods of cancer. ROS mediates various DNA damage and is closely associated with the initiation and development of cancer [40]. However, ROS can induce cell apoptosis and death, which can be used as an important prescription for cancer treatment [41,42,43]. Currently, considerable methods have been developed to improve the level of ROS to induce cancer cell death, and some chemical compounds have been approved as anticancer drugs [44]. Mounting literature has reported that the level of intracellular ROS could be increased by the stimulation of heat stress [45,46]. Consistently, our experiment observed that intracellular ROS of 4T1 cells was increased under heat stress. Notably, the administration of Yoda1 further increased the level of ROS. To identify the role of ROS in the Yoda1 facilitated cell death, a ROS scavenger, catalase, was used. We observed that catalase ameliorated both the heat stress induced cell death and the aggravation effect of Yoda1. This suggested that ROS plays a pivotal role in the precession of heat stress and Piezo1 activation-associated cell injury. Moreover, 4T1 cells treated with Ca^2+^-free medium and Piezo1 siRNA showed alleviated injury under heat stress, indicating that the elevated intracellular Ca^2+^ is triggered by Piezo1 and is responsible for the increased ROS level and the aggravation effect of Yoda1.

**Figure 7:**
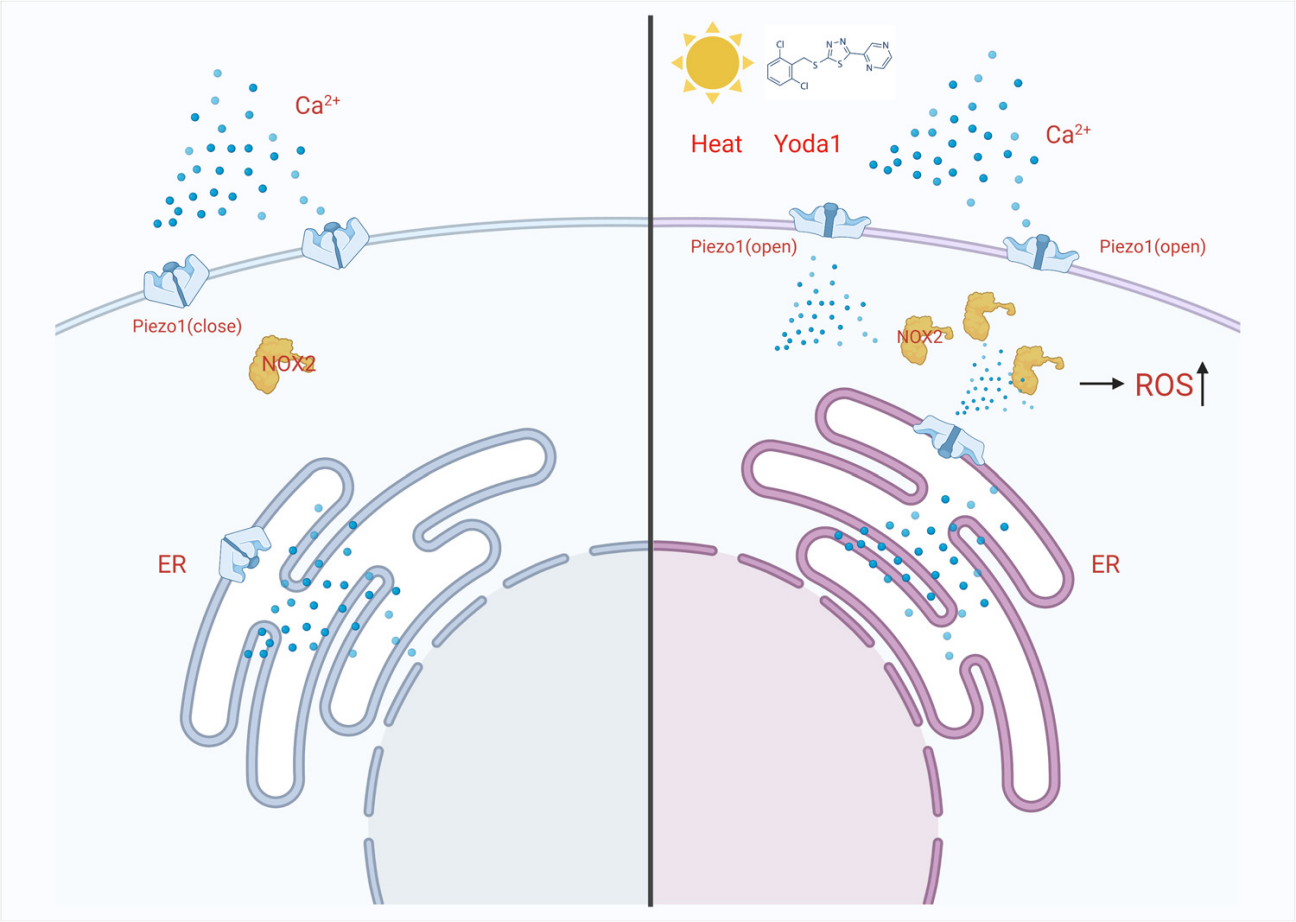
Model of Piezo1-mediated heat stress in photothermal therapy of breast cancer cells. Scheme of Piezo1-mediated heat stress in the breast cancer cell. Photothermal therapy or Yoda1 activates Piezo1, increases the expression of NOX2, and sequentially promotes the release of ROS in the breast cancer cell.

The generation of intracellular ROS is mainly through two pathways. One resource is the mitochondrial respiratory chain, which produces ROS as by-products of cellular metabolism [47]. Another resource relies on the activity of NADPH oxidase (NOX), the main function of this enzyme is ROS production [48]. It is reported that NOX2 is a promising target for cancer therapy [48,49]. In our experiment, heat stress and Piezo1 activation increased the expression of NOX2 in the 4T1 cell. So, we next treated the breast cancer cell with a NOX2 inhibitor (GSK2795039). The results showed that the aggravated cell injury induced by Yoda1 under heat stress was significantly alleviated, which manifested in improved cell viability and declining ROS. Taken together, the elevated ROS induced by heat stress and Yoda1 can be attributable to NOX2 activity. To our knowledge, this mechanism has not been reported in any other literature. After the administration of Ca^2+^-free medium or Piezo1 siRNA, the elevated expression of NOX2 was eliminated under heat stress and Yoda1 treatment. Altogether, heat stress or Yoda1 can activate Piezo1, which increases Ca^2+^ influx and NOX2 expression, leading to increased ROS and cell damage (Figure 7).

We further investigated the potential effect of Piezo1 activation on hyperthermia therapy of breast cancer. The photothermal conversion efficiency of TiCN was determined and the Piezo1-specific agonist significantly enhanced the antitumor effect of TiCN, indicating the excellent potential of Piezo1 activation in breast cancer treatment. Thus, a lot of work is urgently needed in elucidating the biological functions of Piezo1 ion channel in response to heat exposure. In the future, it might be important to alter the regional activity of Piezo1 ion channel in tumor, and the discovery of clinically applicable Piezo1 agonist or antagonist might be of great significance in clinical practice.

## 5 Conclusion

In summary, we proposed a novel target for sensitizing breast cancer to hyperthermia therapy. The activation of Piezo1 ion channel exacerbated heat-induced injury of 4T1 cells, and this property was appropriate for the application of hyperthermia therapy in cancer. The photothermal nanomaterial called TiCN was used to assess the role of Piezo1 activation in PTT of breast tumor. Both the *in vivo* and *in vitro* experiments validated the photothermal conversion efficiency of TiCN and witnessed the efficacy of Piezo1 specific agonist in enhancing the sensitivity of breast tumor to hyperthermia therapy under the 1,064 nm laser irradiation. Mechanistically, Piezo1 activation significantly increased the expression of NOX2 in the breast cancer cells, which may exert the antitumor effect in a ROS-dependent way. Thus, it is imperative to pay more attention to this mechanosensitive ion channel in the setting of heat stress, which may benefit the development of novel medication candidates for antitumor therapy.

## Abbreviations

CCK-8: Cell counting-8
HUVECs: Human umbilical vein endothelial cells
MH: Malignant hyperthermia
NADPH: Nicotinamide adenine dinucleotide phosphate
NOX2: NADPH oxidase 2
PTT: Photothermal therapy
TEM: Transmission electron microscopy
TRPV1: Transient receptor potential vanilloid 1
XPS: X-ray photoelectron spectroscopy
XRD: X-ray diffraction
ROS: Reactive oxygen species
RyR1: Ryanodine receptor 1
